# CONCURRENT CHANGES IN COGNITIVE FUNCTION AND FUNCTIONAL LIMITATIONS: A BIVARIATE LATENT TRAJECTORY ANALYSIS

**DOI:** 10.1093/geroni/igac059.788

**Published:** 2022-12-20

**Authors:** Elizabeth Handing, Stephen Aichele

**Affiliations:** Colorado State University, Golden, Colorado, United States; Colorado State University, Fort Collins, Colorado, United States

## Abstract

With a rapidly aging global population, declines in cognition and functional abilities are an increasingly salient challenge and fear among many older adults. Researchers and clinicians often study changes in cognition and changes in functional limitations as separate trajectories although they frequently co-occur. In this study, we used two-stage structural equation modeling (SEM) to estimate longitudinal trajectories of cognitive and functional limitations using data from the Health and Retirement Study (2010-2016) (N = 12,431, aged 50–91). First, individuals’ longitudinal factor scores (at three measurement occasions, spanning 6 years) were estimated for cognition (indicated by verbal fluency, numeracy, and recall memory) and functional limitations (indicated by difficulties performing daily living activities, instrumental activities, and mobility). A bivariate latent trajectory model was then fit to these scores. Model fit was acceptable (CFI = .91; RMSEA = .036). Parameter estimates showed that, on average, cognition declined slightly (-.08SD/decade) and functional limitations increased gradually (0.38SD/decade). Importantly, individual cognitive declines were significantly correlated with increases in functional limitation (r = -.19, p < .001). This study fills an important research gap by using advanced statistical modeling (bivariate latent growth models) to examine the joint trajectory of cognition and functional decline which underscores the need to consider the interplay of these two abilities. Methods that quantify both individual and group trajectories can aid in better identification of aging patterns and lead to new ways of thinking about interventions to reduce the burden of concurrent decline.

